# 一种新的细胞周期蛋白——Cyclin Y的功能和分子机制的研究进展

**DOI:** 10.3779/j.issn.1009-3419.2013.11.09

**Published:** 2013-11-20

**Authors:** 晓婷 赵, 妹 江, 文涛 岳

**Affiliations:** 101149 北京，首都医科大学附属北京胸科医院细胞室/北京市结核病胸部肿瘤研究所细胞室 Department of Cellular and Molecular Biology, Beijing Tuberculosis and Thoracic Tumor Research Institute, Beijing 101149, China

**Keywords:** 细胞周期素Y, 细胞周期素X, 肿瘤, 分子机制, Cyclin Y, Cyclin X, Tumor, Molecular mechanism

## Abstract

近年来，陆续有一些新的周期素蛋白被发现，细胞周期素Y（cyclin Y, CCNY）即为其中之一。现有的研究表明cyclinY高度保守，在调节细胞周期及转录过程中发挥重要作用，而且cyclin Y在多种肿瘤组织中高表达，在肿瘤增殖调控中可能发挥重要的功能。现就cyclin Y的研究进展及其与肿瘤的关系做一综述。

## Abstract (en)

A novel cyclin, cyclin Y, is one of the most highly conserved members of the cyclin superfamily, which is famous for their important roles in regulating the cell cycle and transcription. Recently, it was found that cyclin Y was up-regulated in many cancers. Cyclin Y may play a crucial role in tumor progression. The function and molecular mechanism of cyclin Y protein will be discussed in this paper.

细胞周期素（cyclin）、周期蛋白依赖性激酶（cyclin-dependent kinase, CDK）、细胞周期蛋白依赖性激酶抑制因子（cyclin-dependent kinase inhibitor, CKI）是细胞周期调控的关键分子，广泛存于真核生物细胞中，它们一起组成细胞周期的调控网络^[1, 2]^。近年的大量研究显示，在细胞发生恶性转化的过程中，除了癌基因与抑癌基因的正负调控之外，细胞周期调控分子也参与了细胞的癌变过程。从一定程度说，肿瘤被认为是一种细胞周期病，肿瘤的发生、发展与细胞周期调控的失调密切相关^[3]^。细胞周期素是一类随着细胞周期变化呈现周期性出现和消失的蛋白，他们的共同特点是具有100个-150个氨基酸组成的周期素盒（cyclin box）保守序列，不同周期素可利用该序列结合不同CDKs，控制细胞周期启动和关键监测点（check point），在细胞周期的不同阶段发挥作用^[1, 4]^。现有研究^[5]^表明几乎所有已知周期素在在肿瘤发生和发展中都起到关键作用。细胞周期素E（cyclin E）在肺癌、乳腺癌、结直肠癌、肝癌中高表达，而细胞周期素D（cyclin D）与非小细胞肺癌、头颈癌、胰腺癌、胆囊癌、乳腺癌等肿瘤发生相关^[6, 7]^。近年来，陆续有一些新的周期素蛋白被发现，细胞周期素Y（cyclin Y, CCNY）即为其中之一。作为一种新发现的细胞周期素，cyclin Y极有可能在肿瘤的发生发展中具有重要的功能，现就cyclin Y的研究进展及其与肿瘤的关系做一综述。

## 1 Cyclin Y的结构特点

Cyclin Y又称CCNY、cyc-Y、周期素盒蛋白1（cyclin box protein 1）、周期素折叠蛋白1（cyclin fold protein 1）或周期素盒携带蛋白1（cyclin-box carrying protein 1），是近年来新发现的一种细胞周期蛋白。其编码基因*CCNY*定位于10号染色体，全长3, 968 bp，有10个外显子、9个内含子。Cyclin Y蛋白含有341个氨基酸，分子量约为39KD，其中143-243位氨基酸高度保守，是CDK结合结构域，亦称为周期素盒。最近，研究者发现了cyclinY的一种异构体——cyclin X（CCNX），除了缺失cyclin Y N末端54个氨基酸残基外，其余蛋白序列与Cyclin Y序列完全相同^[8]^。

## 2 Cyclin Y的亚细胞定位

Cyclin Y的N端第二位氨基酸即甘氨酸为豆蔻酰化位点，将cyclin Y定位于细胞膜，而cyclin X的N末端由于缺少豆蔻酰化位点，在细胞中主要定位于细胞核^[8]^。中国科学院上海生化细胞所的研究人员成功将*CCNY*基因及其N端突变体克隆至真核表达载体pEGFP-N3、pEGFP-C2、pcDNA3-2HA和pMycN3中，发现野生型cyclin Y在半转化型细胞HEK293T细胞中定位于细胞膜，N端豆蔻酰化位点突变的突变体在HEK293T细胞中定位于细胞浆和细胞核中^[9]^，这与Mikolcevic等^[10]^的研究发现是一致的。另外，Li^[8]^等将目的基因CCNX克隆至真核表达载体pCMV-Myc中，发现CCNX的N末端缺少豆蔻酰化位点在人肺癌细胞系H1299中主要定位于细胞核。在我们的研究中也得到了类似的结果，人肺癌细胞系H1299中外源性CCNX主要在细胞核表达。大量的研究显示蛋白质的功能与其亚细胞的定位密切相关。例如，Lonardod等^[11]^发现在非小细胞肺癌中Maspin的胞质定位提示预后不良，若在细胞核内出现则预后较好。但是cyclinY与cyclin X之间的关系以及两者在细胞中的内源性表达情况及定位尚不明确。

## 3 Cyclin Y的功能与分子机制

细胞周期（cell cycle）是指细胞从第一次分裂结束产生新细胞到第二次分裂结束所经历的全过程，分为间期与分裂期两个阶段。间期又分为三期、即DNA合成前期（G_1_期）、DNA合成期（S期）与DNA合成后期（G_2_期）。M期即为细胞分裂期。细胞周期受到由细胞周期蛋白（也称为周期素）、细胞周期蛋白依赖激酶和CDK抑制物构成的网络系统调控，该系统针对多个细胞周期监测点严格控制细胞周期的运行^[1, 2]^。例如cyclin D激活CDK4或CDK6掌控G_1_期的细胞生长^[12, 13]^；Cyclin E作为CDK2的一个正向的调节亚单位，促使细胞进入S期^[12, 14]^。现有的研究表明，细胞周期内有两个阶段最为重要即G_1_期到S期和G_2_期到M期。细胞周期的运行主要依赖于CDK，该酶通过与细胞周期蛋白相结合产生活性，促进细胞周期中G_1_期到S期和G_2_期到M期的转变。

现有的研究^[15]^表明cyclinY高度保守，在调节细胞周期及转录过程中发挥重要作用。但是关于cyclin Y的功能和作用机制尚不清楚。目前*CCNY*基因功能在果蝇中研究比较清楚。在果蝇中*CCNY*基因命名为CG14939，在果蝇的胚胎发生中具有重要的作用，*Cyclin Y*基因突变后会导致果蝇生长迟缓，发育产生缺陷或畸形。Liu等^[16]^采用免疫共沉淀技术鉴定出Cyclin Y与果蝇Ecdysone-induced protein 63E（Eip63E）结合；*Eip63E*基因编码5种高度相关、且具有CDK同源结构域的蛋白亚型, 是果蝇中与cyclin Y结合的CDK。Davidson等^[17]^研究证实，膜定位的Cyclin Y与Eip63E结合，通过磷酸化LRP6 1490位丝氨酸活化Wg/Wnt信号通路，促使果蝇细胞通过G_2_期/M期检查点进入M期。通过NCBI数据库对Eip63E的cDNA序列进行比对，发现人类基因组中基因序列与*Eip63E*基因序列同源的有PCTAIRE protein kinase 1（PCTK1）、PCTK2、PCTK3、PFTAIRE protein kinase 1（PFTK1）、PFTK2、CDK5等。PCTAIRE激酶（PCTAIRE kinases, PCTKs）与PFTAIRE激酶（PFTAIRE kinases, PFTKs）属于高度保守的丝氨酸/苏氨酸激酶，在真核生物中高度保守，具有CDK2激酶结构域的同源序列, 是近年来发现具有CDK功能的激酶，属于cdc2相关的激酶，又名为CDK14^[18, 19]^。与哺乳动物中，PFTAIRE具有Eip63E与cyclin结合的α-螺旋区的氨基酸序列的同源区，因此认为PFTAIRE是Eip63E的同源CDK^[16]^。Gary Davidson团队的研究发现，在果蝇中，PFTK1可以使Wnt受体之一LRP6的1490位丝氨酸磷酸化，从而参与Wg/Wnt信号通路的活化^[17]^。

酵母双杂交的研究结果说明，PFTK1可以与CCNY相互作用，而亚细胞定位分析说明细胞中CCNY的一种异构体定位于细胞膜上，因此，细胞膜上的CCNY可能通过与PFTK1的结合，将PFTK1固定在膜上，引起细胞膜蛋白LRP6的磷酸化^[17]^。作为细胞周期蛋白，CCNY在细胞中的水平随细胞周期的变化而变化，在G_2_/M期水平达到最高；而LRP6 1490位丝氨酸的磷酸化水平也在G_2_/M期达到最高，这充分说明CCNY极可能通过PFTK1参与了Wg/Wnt信号通路的活化。但是，CCNY参与人类细胞周期调控的机理仍不完全清楚，胞浆分布的PFTK1如何转至细胞膜与CCNY结合是需要解决的关键问题。而且，在人类细胞中，CCNY是否能够与其他的CDK结合尚不清楚。

最近，Mikolcevic等通过免疫共沉淀及酵母双杂交的方法证实另一个与Cyclin Y相作用的CDK，即PCTAIRE 1（PCTAIRE kinases 1, PCTK1)，又名CDK16，它与CDK14序列上高度同源；Cyclin Y依赖磷酸化作用与PCTK1相结合并使其共定位于细胞膜；另一方面，Cyclin Y增强了PCTK1激酶的活性，进而促进精子的形成^[10, 20]^。

## 4 Cyclin Y与肿瘤

关于Cyclin Y在肿瘤中的功能及作用机制的报道比较少，与之相关的研究尚处于起步阶段。根据已有的研究，CCNY与CCNX在肿瘤中的作用与分子机制可能有所不同。中科院上海生命科学研究院Jiang等利用Northern blot检测了多种人体组织和细胞系，发现其中至少存在两种Cyclin Y转录本，分别为4 kb和2 kb，4 kb转录本在所有检测的组织和细胞系中低水平表达，2 kb转录本则在睾丸等细胞分裂旺盛的细胞中高表达，在骨骼肌、脑等组织中低表达，而在正常肺组织中无表达^[9]^。这说明2 kb Cyclin Y转录本更能够促进肿瘤的发展，在肿瘤增殖调控中可能发挥重要的功能。但是，由于CCNX与CCNY相比，只是在N端缺少了54个氨基酸，已有的抗体的抗原表位均位于CCNY与CCNX的共有序列，所以在蛋白检测方面很难将CCNY与CCNX区分开；而且，在已有的报道中，多应用RNA干扰技术（RNA interference, RNAi）对cyclin Y的功能进行研究，而RNA干扰的靶点也位于CCNY与CCNX的共有序列，所以难以将CCNY与CCNX的功能进行区分，大多数报道尚未对二者进行区分。

作者前期的研究结果发现*CCNY*基因在非小细胞肺癌肿瘤组织（*n*=60）和肺癌细胞系（*n*=12）中高表达，*CCNY*基因的表达与非小细胞肺癌的组织类型以及肿瘤的大小具有统计学差异；利用RNA干扰技术抑制*CCNY*基因的表达可显著抑制H1299、95D肺癌细胞增殖的速度及小鼠体内成瘤的效率；*CCNY*基因沉默后肺癌细胞周期阻滞于G_1_/S期，从而显著抑制肺癌细胞的体内外生长^[21, 22]^。同样，在直肠癌的研究中也有类似的结果，Ying-Tao等^[23]^通过质谱分析的方法筛选出Cyclin Y蛋白在转移性的人直肠癌细胞系中显著高表达；而且，高表达Cyclin Y能促进直肠癌瘤细胞的细胞增殖、迁移与侵袭，同时在肿瘤细胞的粘附、凋亡及肿瘤的免疫等方面发挥重要的作用，进而可能与直肠癌的远处转移密切相关。另外，Cyclin Y还能促进脑胶质瘤细胞的增殖。利用RNAi抑制CCNY表达可明显抑制脑胶质细胞瘤细胞的增殖、克隆形成以及细胞周期的进程。综上所述，Cyclin Y与肿瘤的发生发展可能密切相关，但是目前Cyclin Y在其它肿瘤中作用研究的很少^[24]^。

另外，研究表明与Cyclin Y结合的CDK PFTK1在肿瘤发生发展过程中可能发生重要的功能。Pang等^[18]^研究发现PFTK1在肝癌组织中高表达，*PFTK1*基因的表达与肿瘤的远处转移及微血管转移密切相关；体外实验亦证实PFTK1可能通过改变F-肌动蛋白的运动进而促进肝癌细胞的迁移与侵袭。Miyagaki等通过免疫组化的方法检测了223例食管鳞状细胞癌组织中PFTK1蛋白的表达，发现PFTK1蛋白不但在食管癌组织中高表达而且与食管癌的预后也密切相关；PFTK1过表达者的生存率明显低于低表达者，同时对化疗的耐药性也显著提高，提示其可作为有效的预后及化疗疗效观察的因子^[25]^。这也说明Cyclin Y在肿瘤进展中极有可能具有重要的作用。

## 5 应用前景

目前越来越多的研究证实Cyclin Y作为细胞周期相关蛋白，其基因和编码蛋白的异常，在胚胎发育、细胞周期进程及肿瘤发生发展中扮演重要的角色。但是目前对Cyclin Y的研究尚少，还存在不少的问题。例如：Cyclin Y在其它肿瘤中表达情况尚不明确；Cyclin Y与其它肿瘤相关基因的相互作用机制尚未十分清楚；Cyclin Y与肿瘤转移的相关性还有待进一步明确；Cyclin Y在肿瘤中的功能与作用机制还亟需进一步的探索和研究。深入研究Cyclin Y不仅有助于揭示肿瘤的发生机制，并可为肿瘤的防治提供一个新的视点。
